# Pharmacogenomics of EGFR-targeted therapies in non–small cell lung cancer: *EGFR* and beyond

**DOI:** 10.1186/s40880-015-0007-9

**Published:** 2015-04-08

**Authors:** Christopher Delaney, Samuel Frank, R Stephanie Huang

**Affiliations:** Biological Sciences Division, University of Chicago, Chicago, IL 60637 USA; Department of Medicine, University of Chicago, 900 E 57th street, KCBD room 7148, Chicago, IL 60637 USA; The Affiliated Hospital, School of Medicine, Ningbo University, Ningbo, Zhejiang 315211 P. R. China

**Keywords:** Pharmacogenomics, Non–small cell lung cancer (NSCLC), Epidermal growth factor receptor (EGFR), Cetuximab, Erlotinib

## Abstract

Commonly observed aberrations in epidermal growth factor receptor (EGFR) signaling have led to the development of EGFR-targeted therapies for various cancers, including non–small cell lung cancer (NSCLC). *EGFR* mutations and overexpression have further been shown to modulate sensitivity to these EGFR-targeted therapies in NSCLC and several other types of cancers. However, it is clear that mutations and/or genetic variations in *EGFR* alone cannot explain all of the variability in the responses of patients with NSCLC to EGFR-targeted therapies. For instance, in addition to *EGFR* genotype, genetic variations in other members of the signaling pathway downstream of EGFR or variations in parallel receptor tyrosine kinase (RTK) pathways are now recognized to have a significant impact on the efficacy of certain EGFR-targeted therapies. In this review, we highlight the mutations and genetic variations in such genes downstream of EGFR and in parallel RTK pathways. Specifically, the directional effects of these pharmacogenetic factors are discussed with a focus on two commonly prescribed EGFR inhibitors: cetuximab and erlotinib. The results of this comprehensive review can be used to optimize the treatment of NSCLC with EGFR inhibitors. Furthermore, they may provide the rationale for the design of subsequent combination therapies that involve the inhibition of EGFR.

## Introduction

Non–small cell lung cancer (NSCLC) accounts for nearly 80% of all lung cancers and is the leading cause of cancer-related deaths worldwide [1,2]. Moreover, late-stage detection limits the treatment options for many patients with NSCLC given that most cancers have already metastasized at the time of diagnosis [1]. In more than one half of all patients with NSCLC, the aberrant epidermal growth factor receptor (EGFR) signaling contributes to the oncogenic phenotype [1]. More recent attempts to treat NSCLC have thus focused on targeting EGFR in order to abrogate the oncogenic signaling mediated by activating *EGFR* mutations (found in approximately 15% of patients with NSCLC), EGFR overexpression, and/or *EGFR* gene copy number enhancement [3-5]. For example, EGFR inhibition is achieved through two main classes of drugs: tyrosine-kinase inhibitors (TKIs) and monoclonal antibodies. Cetuximab (Erbitux™) is a commonly prescribed monoclonal antibody for the treatment of metastatic NSCLC. Cetuximab inhibits EGFR by binding to its extracellular domain, which then blocks ligand-dependent receptor activation [6]. Although less clearly understood, cetuximab also inhibits EGFR signaling by mediating receptor endocytosis and degradation and thus it also decreases ligand-independent EGFR signaling [7]. On the contrary, erlotinib (Tarceva™) is a frequently prescribed TKI for the treatment of NSCLC. By binding to the intracellular kinase domain of EGFR at the ATP-binding site, erlotinib inhibits kinase activity by blocking ATP hydrolysis [1,8-10].

Pharmacogenomic studies have shown that *EGFR* mutation status is associated with erlotinib efficacy and that EGFR overexpression is associated with patient response to cetuximab and other EGFR-targeted agents [1,11-13]. However, even among patients who are selected for specific treatments based on their somatic *EGFR* mutation status or EGFR expression profile, there remains a notable lack of response to EGFR-targeted therapies in a significant portion of the patient population. For instance, approximately 30% of patients with NSCLC with activating *EGFR* mutations do not respond as expected to TKIs against EGFR [1,14]. Therefore, although *EGFR* status is still an important indicator of patient response to EGFR-targeted therapies, it is clearly not the only gene that influences the therapeutic response. A review of the pharmacogenomics of cetuximab and erlotinib instead reveals that other genetic factors, beyond *EGFR*, influence the efficacy of these agents and can potentially guide the treatment of NSCLC with mutant *EGFR* or EGFR overexpression. In fact, cetuximab serves as a model candidate drug with which to explore the effects of such non-*EGFR* genetic variations on the treatment of NSCLC given the established association between Kirsten rat sarcoma viral oncogene (*KRAS*) mutations and poor efficacy of cetuximab in the treatment of colorectal cancer [15]. Similar non-*EGFR* genetic variations have been implicated in modulating erlotinib efficacy in those patients with NSCLC who harbor activating *EGFR* mutations [1,16,17]. More specifically, recent and compelling evidence now suggests that genetic variations in other members of the signaling pathway downstream of EGFR, and also in the non-EGFR receptor tyrosine kinase (RTK) pathways, can influence responses to cetuximab and erlotinib.

### The EGFR signaling network

**Figure 1:**
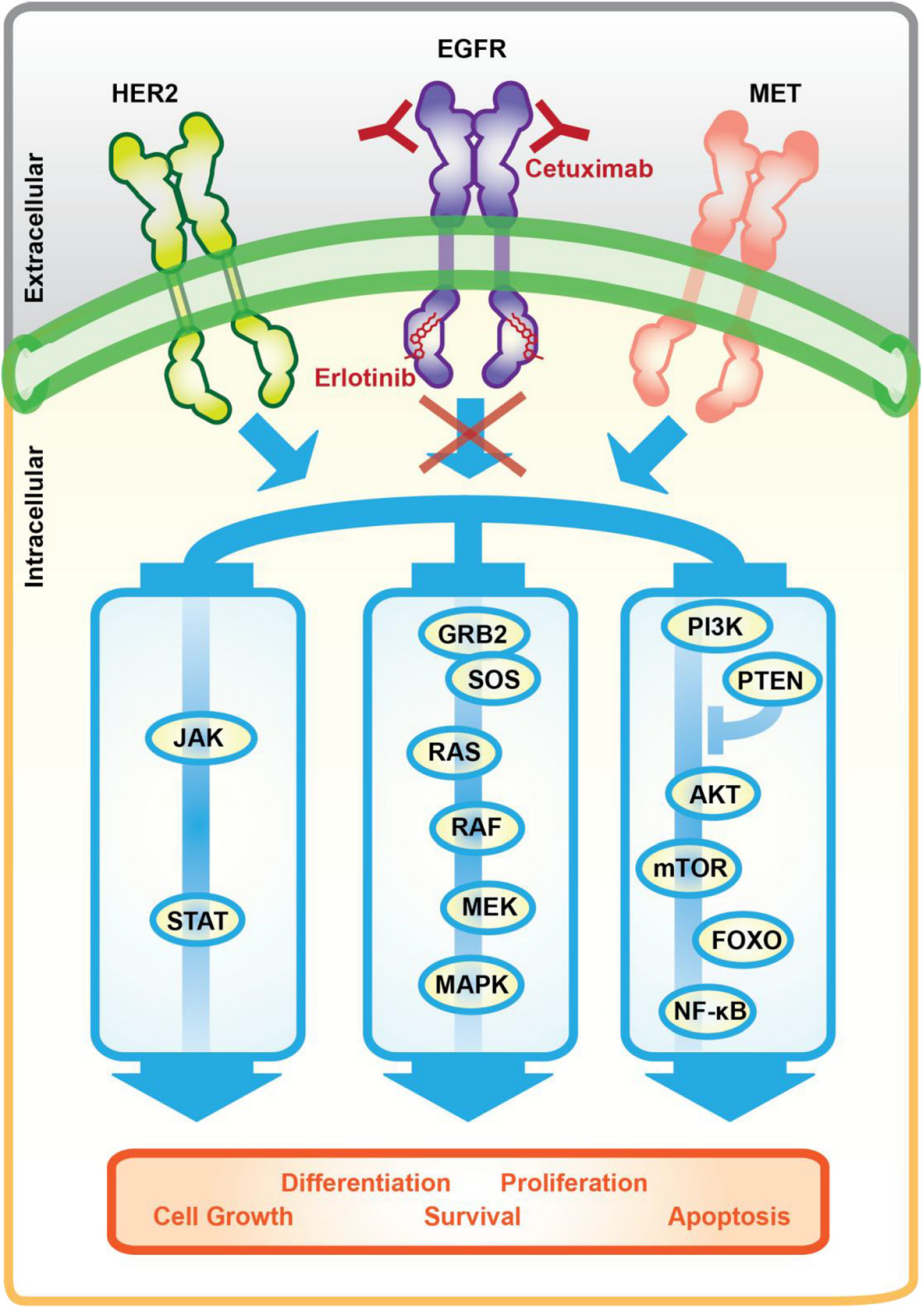
**Schematic representation of the primary epidermal growth factor receptor (EGFR) signaling pathway.** During normal EGFR signaling, receptor activation is dependent on ligand-mediated receptor dimerization. Once the subunits dimerize, a series of phosphorylation events serve to enhance EGFR kinase activity to stimulate the activation of downstream targets. Downstream signals are propagated by EGFR through three central pathways via both the direct phosphorylation of downstream targets (the JAK/STAT pathway) and the membrane recruitment of key adaptor proteins (the PI3K/AKT and MAPK pathways) to promote cell survival and proliferation. The EGFR signaling through a conserved core of three downstream signaling pathways demonstrates how the activation of this pathway via parallel RTKs, such as HER2, HER3, and MET, can circumvent the inhibitory effects of cetuximab and erlotinib on EGFR. EGFR, epidermal growth factor receptor; JAK/STAT, Janus activated kinase/signal transducer and activator of transcription; PI3K/AKT, phosphoinositide 3-kinase/protein kinase B; MAPK, mitogen-activated protein kinase; HER2, human epidermal growth factor receptor 2; HER3, human epidermal growth factor receptor 3; MET, hepatocyte growth factor receptor; SOS, son of sevenless; GRB2, growth factor receptor-bound protein 2; RAS, rat sarcoma family of proteins; RAF, rapidly accelerated fibrosarcoma; MEK, MAPK kinase; PTEN, phosphatase and tensin homolog; mTOR, mammalian target of rapamycin; FOXO, forkhead box proteins; NF-κB, nuclear factor-kappa B.

EGFR signaling contributes to the regulation of fundamental biological processes including cell proliferation, differentiation, survival, adhesion, homeostasis, and tumorigenesis [18-21]. Exceedingly complex and highly regulated signal transduction mechanisms are required to govern such varied EGFR responses to external stimuli [19,20,22]. Given the vast complexity of the EGFR signaling network, it is hardly surprising that genetic factors beyond *EGFR* mutations or variable expression patterns may modulate therapeutic responses to EGFR-targeted agents. Here, we present an overview of EGFR signaling and highlight the primary downstream signaling pathways (Figure 1).

EGFR is a membrane-spanning cell surface receptor and consequently links internal signaling pathways to the extracellular environment [19]. When stimulated by extracellular ligand binding, EGFR can initiate intracellular kinase cascades through its intracellular tyrosine kinase domain (Figure 1) [18,19]. In humans, 7 peptide growth factors function as EGFR ligands, among which the canonical ligand is epidermal growth factor (EGF) [18,21]. These ligands can either be membrane-bound to adjacent cells or soluble if they undergo proteolysis at the cell membrane, which allows EGFR to integrate stimuli from a variety of local and distant sources (Figure 1) [21,23].

Structurally, EGFR has a domain architecture that consists of four extracellular domains, one transmembrane domain, one juxtamembrane domain, and one tyrosine kinase domain followed by a flexible C-terminal tail (Figure 1) [24]. Prior to ligand binding, EGFR exists as a catalytically inactive monomer in the cell membrane [19,24]. Following bivalent ligand binding to a single EGFR molecule, the receptor changes conformation to facilitate dimerization and activation [18,19,24]. Receptor activation then proceeds through a defined series of auto and transphosphorylation events between the kinase domains in the EGFR dimer [19]. The activated kinases further proceed to phosphorylate the C-terminal tail regions on the neighboring receptor, creating phosphorylated tyrosine (pY) residues [19,25]. The pY residues then serve as docking sites for proteins that are capable of binding pY motifs (Figure 1) [19,20,26]. The docked proteins can then either transduce intracellular signals following direct phosphorylation by EGFR or initiate higher-order signaling cascades through scaffolding and localization mechanisms (Figure 1) [19,27,28].

Once activated, EGFR transduces its numerous cellular responses through three primary signaling cascades: the mitogen–activated protein kinase (MAPK) pathway, the phosphoinositide 3-kinase/protein kinase B (PI3K/AKT) pathway, and the Janus kinase/signal transducer and activator of transcription (JAK/STAT) pathway (Figure 1) [1,18,25]. Notably, these three central signaling pathways, although downstream of EGFR, are also activated by almost every other RTK (Figure 1) [19,29]. Once EGFR activates the rat sarcoma (RAS) family of proteins, the MAPK pathway proceeds through the consecutive phosphorylation of rapidly accelerated fibrosarcoma (RAF), MAPK kinase (MEK), and MAPK via the recruitment of the son of sevenless/growth factor receptor-bound protein 2 (SOS/GRB2) complex (Figure 1) [18,30]. The MAPK cascade results in the activation of various transcription factors, which act to alter cellular expression profiles and to promote proliferation (Figure 1) [30]. The PI3K/AKT pathway not only governs the anti-apoptotic and pro-survival signals associated with EGFR activation but also plays a role in the regulation of cell growth via the activation of the mammalian target of rapamycin (mTOR) (Figure 1) [1,31]. Moreover, AKT activation promotes the accumulation of nuclear factor-kappa B (NF-κB) in the nucleus, which further promotes cell survival [32]. The PI3K/AKT pathway is also negatively regulated by phosphatase and tensin homolog (PTEN), a phosphatase that opposes PI3K activity to repress AKT activation (Figure 1) [33]. JAK/STAT signaling is initiated when proteins of the JAK family are recruited to EGFR and are activated via phosphorylation [34]. Activated JAK proteins can phosphorylate proteins of the STAT family, which then dimerize, translocate to the nucleus, and enact global cellular effects, particularly those related to cell survival and proliferation (Figure 1) [34]. Clearly, the downstream effects of EGFR signaling reveal the oncogenic potential of aberrant EGFR signaling.

## Review

### Erlotinib and cetuximab as EGFR-targeted therapeutics in NSCLC: pharmacogenomics of *EGFR*

The genetic variation in *EGFR* and how this variation alters typical EGFR signaling, plus its effects on cetuximab and erlotinib efficacy, have all been extensively reviewed elsewhere [1,35,36]. However, it is important to briefly describe the genetic variations in *EGFR* in order to provide a framework for the interpretation of how non-*EGFR* genetic variations can affect responses to cetuximab and erlotinib; namely, aberrant activation of signaling pathways downstream of, or parallel to, the EGFR signaling network can compensate for EGFR inhibition. On the most basic level, two types of genetic variations in *EGFR* drive hyperactive signaling: constitutive activation and overexpression [1,36]. EGFR molecules can be constitutively active due to either point mutations in the kinase domain or larger deletions in the juxtamembrane domain that relieve the autoinhibition of the ligand-free monomer [1,19,36,37]. Thus, even in the absence of ligand, these receptors are active and continually promote downstream signaling. EGFR overexpression is another mechanism that contributes to hyperactive EGFR signaling. The observed increase in signaling strength from EGFR overexpression is partly due to the increase in the number of receptors, which amplify the downstream signaling effects [1,36,38]. In addition, the overexpression of wild-type (wt)-*EGFR* can promote rogue signaling through a ligand-free activation mechanism comparable to that of mutant *EGFR*. Specifically, the increased local concentration of EGFR at the cell membrane can increase the stochastic, ligand-free dimerization of EGFR that activates the receptors [39].

Unlike newer TKIs that irreversibly bind to EGFR, erlotinib reversibly binds to the ATP-binding pocket of EGFR and inhibits catalytic activity via the prevention of ATP-dependent activation of downstream targets [8,40]. Moreover, erlotinib is very similar to gefitinib, another commonly studied EGFR TKI, in that they have similar mechanisms of action and have been demonstrated to have similar effectiveness in the treatment of NSCLC, despite reports of minor differences in toxicity [41,42]. Given its mechanisms of action and the prerequisite of ATP hydrolysis for EGFR catalytic activity, erlotinib was predicted to elicit a positive drug response in patients with NSCLC as long as they were positive for EGFR expression, regardless of the *EGFR* mutation status. However, a wealth of research has been conducted that suggests that only certain activating mutations in *EGFR* serve as positive clinical indicators for the use of erlotinib in patients with NSCLC [25,43-46]. Surprisingly, it has been demonstrated that while several mutations in *EGFR* confer a susceptibility to erlotinib, others facilitate the opposite effect—a resistance to erlotinib [47-49]. The most common activating mutations in *EGFR*, which account for 90% of all *EGFR* mutations in cases of NSCLC, are exon 19 deletions and the L858R mutation in the tyrosine kinase domain (Table 1) [1]. Other point mutations such as G719C, G719S, G719A, and L861Q similarly result in mutant forms of *EGFR* that are hyperactive (Table 1) [1]. Importantly, all of these activating mutations have been shown to incur sensitivity to EGFR TKIs such as erlotinib (Table 1) [1]. Other activating *EGFR* mutations, primarily in exon 20, such as T790M (gatekeeper mutation), L747S, D761Y, and T854A, also result in overactive EGFR signaling but instead grant resistance to erlotinib and other TKIs that are similar to erlotinib [1,48,50,51]. More importantly, given their association with acquired TKI resistance, these mutations appear to overrule the normal erlotinib sensitivity incurred by the more common EGFR-activating mutations when both mutation types are concurrently present (Table 1) [1,48]. As a consequence, several irreversible potent anti-EGFR agents have either been approved by the United States Food and Drug Administration (FDA) (e.g., afatinib) or show promise in clinical trials to overcome the T790M-mediated EGFR TKI resistance [52-55]. The variability in the response to erlotinib in the treatment of NSCLC is further accentuated by the fact that only approximately 10% of patients without activating *EGFR* mutations demonstrate a drug response to first-line erlotinib treatment [1,56-58]. Clearly, the gene-drug relationship of *EGFR* and erlotinib is dependent upon more than *EGFR* mutation status alone, and these findings suggest the need to elucidate the predictive value of other specific, non-*EGFR* genetic variants that could guide the treatment of NSCLC.

**Table 1:** **Pharmacogenomics of cetuximab and erlotinib: potential biomarkers that are predicative of drug efficacy**

**Biomarker**	**Cetuximab**	**Erlotinib**
**EGFR biomarkers**		
***EGFR***	Expression profile that incurs sensitivity:	Mutations that incur sensitivity [1]:
	♦High EGFR expression (as determined by IHC in NSCLC [11,13,60,61] and flow cytometry in colorectal cancer [12])	♦Exon 19 deletions
		♦L858R
		♦G719X (X = C, S, or A)
		♦L861Q
	Expression profile that incurs resistance:	Mutations that incur resistance (primarily exon 20 insertions) [1]:
	♦Low EGFR expression (as determined by IHC) [6,11,13,60,61]	♦T790M
		♦L747S
		♦D761Y
		♦T854A
**Biomarkers in downstream components of the EGFR signaling**		
***KRAS***	Little predictive value in NSCLC [16,68]	Mutations that incur resistance (exon 1 point mutations) [1,17,66] :
		♦G12X
		♦G13X
***PIK3CA***	Mutations that incur resistance (in colorectal cancer and human head and neck cancer)	Activating mutations that incur resistance [72,73,76]
	Exon 20 kinase domain mutations:	Exon 9 helical domain mutations:
	♦H1047X (primarily H1047R) [77-79]	♦E542X (primarily E542K)
		♦E545X (primarily E545K)
		Exon 20 kinase domain mutation:
		♦H1047X (primarily H1047R)
***PTEN***	Mutations that incur resistance:	Mutations that incur resistance [80]:
	♦Homozygous *PTEN* deletions	♦Homozygous *PTEN* deletions
	♦Missense/loss of function mutations [114]	♦Missense/loss of function mutations
	Expression profile that incurs resistance:	Expression profile that incurs resistance:
		♦Low/null *PTEN* expression [81,82]
	♦Null *PTEN* expression [83,84]	Expression profile that incurs sensitivity:
		♦High *PTEN* expression [81]
***NF-***κ***B***	Expression profile that incurs sensitivity:	Factors that enhance sensitivity:
	♦Low *NF-*κ*B* (as determined by evidence from patients with colorectal cancer [88])	♦Low *NF-*κ*B* expression (via knockdown of RELA subunit) [87]
	Expression profile that incurs resistance:	♦Inactive *NF-*κ*B* associated with increased inhibitor of *NF-*κ*B* (*NFKBIA*) expression [87]
	♦High *NF-*κ*B* (as determined by evidence from patients with colorectal cancer [88])	Factors that enhance resistance:
		♦High *NF-*κ*B* (as determined by *NF-*κ*B* overexpression)
		♦Overactive NF-κ*B* (via *NFKBIA* knockdown [87], IKK overexpression [87], and high levels of AEG-1 [1])
**Biomarkers in other RTKs**		
***MET***	Incurred resistance from:	Incurred resistance from:
	♦*MET* amplification [98]	♦*MET* gene amplification [92,93,96]
	♦MET activation [98,99]	♦Enhanced MET signaling dependent on HER3 [97]
		♦Increased concentrations of HGF ligand [94]
***HER2***	Incurred resistance from:	Incurred resistance from:
	♦Enhanced copy number of *HER2* gene [103]	♦Enhanced copy number of *HER2* gene [103]
	♦HER2-mediated signaling [101,103]	♦Activating *HER2* mutations in exons 18–21 (kinase domain), notably 12-bp duplication/insertion of YVMA in exon 20 at codon 776 (HER^YVMA^) [103]
***HER3***	Incurred resistance from:	Incurred sensitivity from:
	♦Enhanced EGFR/HER3 activity via up-regulated *HER3* [101]	♦Low *HER3* expression [115]
	♦Dimerization and transactivation of HER2/3 in an EGFR-dependent manner [101]	♦Down-regulation of *HER3* by RNAi in *EGFR*-mutant and wild-type *EGFR* cell lines [97]
	Restored sensitivity from:	
	♦Down-regulation of *HER3* by siRNA [101]	

*EGFR*, epidermal growth factor receptor; *KRAS*, Kirsten rat sarcoma oncogene; *PIK3CA*, phosphatidylinositol-4,5-bisphosphate 3-kinase, catalytic subunit alpha; *PTEN*, phosphatase and tensin homolog; *NF-*κ*B*, nuclear factor kappa-B; RTK, receptor tyrosine kinase; *MET*, hepatocyte growth factor receptor; *HER2/3*, human epidermal growth factor receptor 2/3; siRNA, small interfering RNA; IHC, immunohistochemistry; NSCLC, non–small cell lung cancer; RELA, rel homology domain A; *NFKBIA*, nuclear factor of kappa light polypeptide gene enhancer in B-cell inhibitor, alpha; IKK, IκB kinase; *AEG*-1, astrocyte elevated gene-1; HGF, hepatocyte growth factor; RNAi, RNA interference.

Some studies suggest that the response to erlotinib is also improved in patients with EGFR overexpression [1,59]. However, this association might be due to the link between the efficacy of erlotinib and *EGFR* mutation status because activating *EGFR* mutations often promote increased expression and altered copy number of *EGFR*. Moreover, other studies have provided contrary evidence with regard to the association between EGFR expression and benefits from erlotinib treatment [1]. Unlike erlotinib, cetuximab has an efficacy that has been associated with high levels of *EGFR* (mutant or wild-type) expression but not with *EGFR* mutations or copy number variation (Table 1) [11,13]. Variation in *EGFR* expression alone, though, is not sufficient to explain the pharmacogenomics of cetuximab in the treatment of NSCLC. For example, one trial evaluated the expression of immunohistochemically detectable EGFR as a biomarker, and recruited patients to examine the effectiveness of cetuximab in combination with first-line chemotherapy [60,61]. The trial showed that standard chemotherapy plus cetuximab was superior to chemotherapy alone in patients whose tumors expressed EGFR [60,61]. In contrast to these findings, the analysis of a similar trial that lacked patient selection based on EGFR expression status found no association of EGFR expression status with cetuximab benefit; however, this study confirmed that *EGFR* mutation status and gene copy number are not predictive biomarkers of the response to cetuximab treatment [60-63]. Despite these inconsistencies, a meta-analysis of 4 such trials has provided evidence that a high level of EGFR expression can predict clinical benefits from cetuximab therapy [13]. Therefore, unlike *EGFR* mutation status for erlotinib, the most appropriate biomarker for the selection of patients with NSCLC for cetuximab treatment appears to be the expression level of EGFR. However, further studies are still needed to corroborate these findings given the debate over the cutoff between high and low expression and because several studies still question the predictive value of the expression level of EGFR in the treatment of patients with NSCLC [6,64,65].

### Pharmacogenomics beyond *EGFR:* downstream pathway members

Clear pharmacogenomic associations exist between cetuximab and erlotinib and genes other than *EGFR*. Some of the most well-characterized associations involve genetic variations in downstream pathway members in the EGFR signaling network. Given that the signaling pathways that are activated by EGFR primarily consist of kinase phosphorylation cascades, it is not surprising that mutations in these kinases are similar to those that result in the constitutive activation of EGFR. As shown in Figure 1, EGFR regulates numerous signaling pathways after activation and thus mutations in any downstream pathway member could foreseeably compensate for EGFR inhibition via the overstimulation of a specific node of downstream signaling.

Prime examples of genetic variation in signaling proteins downstream of EGFR are *KRAS* mutations (Table 1) [1,16]. *KRAS* mutations have been linked to the reduced efficacy of erlotinib in the treatment of NSCLC and to that of cetuximab in the treatment of colorectal cancer [1,15,17]. Moreover, evidence from other RTK-targeted TKIs suggests that mutations in *KRAS* are highly associated with intrinsic resistance to TKI treatment [1,17]. For instance, one study found that 95% of patients with NSCLC who had detectable *KRAS* mutations were resistant to the treatment with TKIs and showed continued disease progression [1]. In addition, multiple studies have demonstrated that tumors with activating *KRAS* exon 1 mutations at G12X and G13X are associated with a lack of response to erlotinib and gefitinib (Table 1) [17,66]. Notably, resistance to erlotinib does not appear to be modulated by specific subtypes of activating *KRAS* mutations, unlike the variable erlotinib sensitivities incurred by different *EGFR* mutations [67,68]. Granted, while most of the evidence supports that *KRAS* mutations grant resistance to EGFR-targeted TKIs, some debate remains concerning the prognostic value of the *KRAS* genotype on TKI treatment of NSCLC. This is especially true given that in rare cases, patients with *KRAS* mutations still respond to erlotinib [66,69]. Although *KRAS* mutations are generally thought of as mutually exclusive to *EGFR* mutations, overexpression of (wt)-EGFR appears to be associated with a response to erlotinib in these patients with *KRAS* mutants. This could explain the atypical drug sensitivity exhibited by these patients [1,66,69].

With respect to cetuximab for the treatment of colorectal cancer, the negative prognostic value of *KRAS* mutations is rigorously defined and is similar to the lack of a response to erlotinib in *KRAS-*mutant patients with NSCLC [15]. However, current evidence suggests that *KRAS* mutations in patients with NSCLC do not necessarily predict a poor response to cetuximab treatment [16,68]. Instead of contradicting the importance of *KRAS* variability in the treatment of NSCLC, the different prognostic values of *KRAS* mutations in colorectal cancer versus NSCLC only confirm the need to determine how non-*EGFR* genetic variations affect the outcome of cetuximab treatment. For example, one convincing explanation for the variable responses to cetuximab in patients with *KRAS*-mutant colorectal cancer compared with patients with *KRAS*-mutant NSCLC is that the *KRAS* mutations found in each cohort are different and could thus elicit variable protein-level effects on the structure and function of KRAS. More specifically, a significantly larger number of KRAS-activating G > T DNA transversions were found at codon 12 of the *KRAS* gene in patients with NSCLC compared with those found in patients with colorectal cancer [68,70]. This might be due to the association of such DNA transversion events with tobacco-related carcinogens [16,68,70]. The *KRAS* transversion mutations in patients with NSCLC correspond to a greater frequency of the G12C variant compared with the more predominant G12D variant found in patients with colorectal cancer [68]. Interestingly, recent findings have shown that in the setting of colorectal cancer, at least 12 subtypes of *KRAS* mutations across codons 12 and 13 associate with variable yet negative efficacy of cetuximab [68,71]. An understanding of what the different *KRAS* mutations are in NSCLC and how they modulate the efficacy of cetuximab could perhaps reveal similar subsets of *KRAS* mutations with specific predictive value to treatment options of NSCLC.

If *KRAS* mutations are the most predominant genetic alterations in the MAPK pathway that influence the efficacy of EGFR-targeted therapies, then variation in the phosphatidylinositol-4,5-bisphosphate 3-kinase, catalytic subunit alpha (*PIK3CA*) is perhaps the corresponding genetic abnormality in the downstream portion of the PI3K/AKT pathway (Table 1) [1]. *PIK3CA* encodes the p110α subunit of PI3K and is thus an integral part of the PI3K/AKT signaling pathway (Figure 1) [1,31]. Previous evidence has shown that *PIK3CA* mutations frequently co-exist with *EGFR* mutations in NSCLC and that these mutations could be predictive of reduced sensitivity to TKIs (Table 1) [1,72-74]. Two of the most frequent mutations in the helical domain of *PIK3CA,* E542K and E545K, as well as one mutation in the kinase domain, H1047R, are known to activate the p110α subunit and to stimulate oncogenic signaling via the PI3K/AKT pathway (Table 1) [73,75]. Notably, when such activating *PIK3CA* mutations are introduced into *EGFR*-mutant lung cancer cells, they impart a partial resistance to TKIs (Table 1) [73-76]. Interestingly, while this *in vitro* association between *PI3KC* mutations and enhanced resistance to EGFR-targeted TKIs has yet to be replicated in the clinic in patients with NSCLC, one recent study confirmed the high frequency of co-mutations of *PI3KC* and *EGFR* in patients with NSCLC [72]. The same study also found that the status of *PI3KC* mutations was a negative prognostic indicator for survival in patients with (wt)-*EGFR* and (wt)-*KRAS* subtypes [72]. Moreover, although limited experimental data are available to explore the exact prognostic value of *PIK3CA* mutations on the efficacy of cetuximab in NSCLC, activating *PIK3CA* mutations in only the p110α catalytic domain, and not in the helical domain, appear to grant similar resistance to cetuximab in colorectal cancer cohorts (Table 1) [77]. Moreover, other studies involving human head and neck cancer cell lines found that treatment with either a PI3K inhibitor or a dual PI3K/mTOR inhibitor enhances the sensitivity of cancer cells to cetuximab *in vitro* and *in vivo* [78,79]. Thus, *PI3K* mutations may contribute to the reduced efficacy of erlotinib and cetuximab in specific subtypes of cancer cells; however, further investigation is needed to confirm these associations in the clinical setting of NSCLC.

Given the important regulatory role of PTEN in AKT activation (Figure 1), it is not surprising that *PTEN* is another gene from the PI3K/AKT pathway with genetic variations that are associated with the efficacy of EGFR-targeted drugs [74]. Namely, one screen of various NSCLC cell lines revealed that the homozygous loss of *PTEN* was associated with the resistance to erlotinib in the H1650 cell line [80]. In agreement with that report, another study found that *PTEN* overexpression was associated with prolonged survival after TKI treatment in patients with NSCLC, which suggests that low expression or deletion of *PTEN* is associated with shortened survival in patients with NSCLC [81]. *PTEN* also plays a role in the modulation of the response to cetuximab. For instance, NSCLC cell lines were found to acquire resistance to cetuximab, as well as to erlotinib, as a result of enhanced PTEN instability and degradation [82]. This finding is notable in that it highlights how, apart from the presence of a clear homozygous *PTEN* deletion, *PTEN* genotyping may be less effective than PTEN expression testing for the determination of actual PTEN levels. Additional studies of the effects of *PTEN* expression on the response to cetuximab in patients with metastatic colorectal cancer corroborate the results in NSCLC in that the loss of *PTEN* expression was found to be associated with poor overall survival and drug resistance during cetuximab therapy [83,84].

Genetic variations in the NF-κB transcription factor (Figure 1), a downstream member of the PI3K/AKT pathway, also associate with specific responses to erlotinib and cetuximab (Table 1) [1]. Specifically, the nuclear factor of kappa light polypeptide gene enhancer in B-cells inhibitor, alpha (*NFKBIA*) gene encodes the inhibitor of NF-κB (IκBα) protein, which inhibits NF-κB activity by binding NF-κB and confining it to the cytoplasm [1,85,86]. Strong evidence supports that the knockdown of *NFKBIA* confers partial resistance to erlotinib in lung cancer cell lines through elevated levels of NF-κB and that this resistance can be reversed by the inhibition of NF-κB or by the overexpression of *NFKBIA* (Table 1) [1,87]. It was also demonstrated that *NFKBIA* expression is a successful prognostic biomarker for patients with *EGFR*-mutant NSCLC who are treated with erlotinib and that low *NFKBIA* expression is predictive of poor progression-free survival and overall survival [1,87]. Notably, *NFKBIA* silencing is also found far more frequently in patients with NSCLC who lack *EGFR* mutations than in those with *EGFR*-mutant NSCLC, which suggests that such downstream activation of the PI3K/AKT pathway can bypass the need for mutations in upstream driver proteins during carcinogenesis [85]. However, the mechanism that surrounds the variable expression of *NFKBIA* has not been extensively studied. Further support for the importance of NF-κB comes from studies on astrocyte elevated gene-1 (*AEG*-1), an oncogene that can increase NF-κB activity by activating the IκB kinase (IKK), a protein that functions to destabilize IκBα (Table 1) [1]. Patients with NSCLC whose tumors express higher levels of AEG-1 appear to have poorer outcomes than those with lower levels of AEG-1 after treatment with TKIs, which suggests that genetic variations in *AEG-1* could alter EGFR-targeted drug response in a similar fashion to *NFKBIA* (Table 1) [1]*.* With respect to cetuximab, while no specific evidence links genetic variations in *NF-*κB or *NFKBIA* with drug efficacy on NSCLC, patients with colorectal cancer whose tumors are negative for NF-κB expression have a positive predictive response to cetuximab and a longer overall survival compared with patients whose tumors are positive for NF-κB expression; the results were similar after erlotinib treatment in patients with NSCLC (Table 1) [1,88].

### Pharmacogenomics beyond *EGFR:* Non-EGFR RTKs

Just as genetic variations in the members of the EGFR signaling pathway can bypass EGFR inhibition when downstream effectors of EGFR are activated, mutations in, or the overexpression of, non-EGFR RTKs can also thwart the efficacy of EGFR-targeted agents via the activation of parallel signaling pathways. While the term “parallel” is used to signify that a different RTK initiates the signaling cascade, it is important to emphasize that almost all RTKs function to activate the same small number of central pathways (Figure 1) [29]. Therefore, downstream of RTK activation, these parallel pathways merge with the previously described pathways downstream of EGFR (Figure 1). Significantly, there are only 58 known RTKs among the 90 tyrosine kinases in the human genome [19,89]. Given the redundancy of the pathways downstream of activation, aberrant activation of any other RTKs could foreseeably hinder a patient’s response to erlotinib or cetuximab. This can therefore provide a mechanism for both primary and acquired resistance to these EGFR-targeted agents.

EGFR-targeted therapies in NSCLC have been linked to genetic abnormalities in the *MET* gene, which encodes the MET protein or hepatocyte growth factor receptor (HGFR), an RTK that recognizes the hepatocyte growth factor (HGF) ligand [90]. In lung cancer, various mutations in both the juxtamembrane domain and the extracellular domains of *MET*, along with *MET* amplification, are known to activate the receptor and stimulate the PI3K/AKT signaling [91]. For instance, one study found that increased *MET* copy number and MET overexpression are negative prognostic factors for surgically resected NSCLC (Table 1) [1,92]. In addition, *MET* gene amplification and overexpression were found to be associated with resistance of NSCLC cell lines to both erlotinib and gefitinib (Table 1) [93]. MET activation via HGF ligand stimulation has also been shown to induce resistance to EGFR-targeted TKIs even in the presence of activating *EGFR* mutations (Table 1) [94]. Notably, gene amplification of *MET* has been identified in up to 20% of *EGFR*-mutant tumors that have been pretreated with EGFR-targeted TKIs (Table 1). This suggests a role for MET in the mediation of acquired resistance to these drugs since *MET* amplification was rarely concomitant with the common resistance-granting T790M *EGFR* mutation [1,76,91,95,96]. Furthermore, one study detailed a mechanism for such *MET-*mediated resistance by showing that an EGFR TKI-sensitive lung cancer cell line can develop resistance to gefitinib as a result of *MET* amplification via human epidermal growth factor receptor 3 (HER3)-dependent activation of PI3K (Table 1) [97]. Similarly, while the associations between cetuximab and *MET* are currently limited to the setting of colorectal cancer, *MET* amplification and activation were associated with acquired resistance to cetuximab in treated patients, and cetuximab-induced MET activation was found to contribute to cetuximab resistance in certain colon cancer cell lines (Table 1) [98,99].

Beyond its role in the modulation of overactive MET signaling, HER3, a kinase-impaired RTK in the EGFR family, can form active heterodimers with other members of the EGFR family, namely EGFR and human epidermal growth factor receptor 2 (HER2) [1,100]. It thus stands to reason that *HER3* expression patterns and mutation status could impact the efficacy of therapies based on EGFR inhibition. Indeed, one study showed elevated levels of not only MET but also HER2 and HER3 in cells that developed an acquired resistance to cetuximab [101]. The authors propose that cetuximab treatment induces the up-regulation of *EGFR*, which allows EGFR to form heterodimers with HER3, HER2, and MET. This allows for the maintenance of downstream EGFR signaling despite the inhibition of EGFR homodimers [101]. The same study further supports such a heterodimer-mediated mechanism of resistance by demonstrating strong anti-proliferative effects when these cancer cells were treated with both an inhibitor of HER2/3 heterodimerization and cetuximab, which suggests that HER2/3 heterodimers also help mediate cetuximab resistance in an EGFR-dependent manner (Table 1) [101]. Given that HER2 is the preferred dimerization partner of HER3 and that HER2/3 heterodimers are more active and have greater oncogenic potential than other dimers of the EGFR family, it is logical that HER2/3 dimers form and drive rogue signaling once both HER2 and HER3 are recruited to the membrane by EGFR [102]. Therefore, such findings detail one method, similar to those described for MET-mediated resistance, by which cells can evade EGFR inhibition from cetuximab. This is primarily accomplished by the re-establishment of downstream tumorigenic signaling via coordination with alternative RTKs.

The trend of up-regulated RTK activity that modulates resistance to EGFR-targeted therapies is also observed with *HER2.* For example*,* enhanced copy number of the *HER2* gene is associated with increased resistance to both erlotinib and cetuximab (Table 1) [103,104]. Moreover, activating *HER2* mutations are similarly associated with poor response to erlotinib, even when they are concurrent with EGFR-sensitizing mutations (Table 1) [103]. Such mutations are typically found in exons 18–21 of *HER2* and alter the tyrosine kinase domain, which activates the receptor in the absence of ligand [103]. The most common *HER2* mutation is a 12-bp insertion at exon 20, codon 776, which duplicates the amino acid sequence YVMA (HER^YVMA^) and thereby alters the HER2 ATP-binding pocket [103]. HER^YVMA^ can activate EGFR in the absence of EGFR ligands to bypass sensitivity to erlotinib and other EGFR-specific TKIs (Table 1) [103]. However, because mutant *HER2* compensates for EGFR inhibition in cells that harbor HER^YVMA^, these cells are instead sensitive to HER2 and dual HER2/EGFR inhibitors, which suggests alternative therapeutic approaches for individuals with NSCLC who harbor mutations in *HER2* [103].

Our review focuses on parallel pathways that involve MET, HER2, and HER3; however, other RTKs activate the same downstream signaling cascades and thus could also mediate responses to EGFR inhibition. Namely, the vascular endothelial growth factor receptor (VEGFR), the fibroblast growth factor receptor (FGFR), the platelet-derived growth factor receptor (PDGFR), the AXL/UFO receptor tyrosine kinase, the insulin-like growth factor 1 receptor (IGF1R), and still other RTKs similarly appear to contribute to the observed resistance to EGFR inhibition in NSCLC [105-109]. Nevertheless, our summarized findings highlight the clinical value of the assessment of the genetic variation in RTKs beyond EGFR that have the potential to activate oncogenic pathways despite the presence of EGFR inhibitors. Our findings also emphasize mechanisms of both primary and acquired resistance; specifically, mutations or aberrations in other RTKs can exist concurrently with *EGFR* mutations and can even be present prior to EGFR-targeted therapy.

## Discussion and conclusions

Focused pharmacogenomics research has discovered much about *EGFR* and how *EGFR* mutations and expression can guide EGFR-targeted treatment options in patients with NSCLC. The genetic variations in *EGFR*, however, are unable to accurately predict drug responses to EGFR-targeted agents for all patients with hyperactive EGFR signaling, particularly during treatment with erlotinib and cetuximab (Table 1). In patients with mutant *EGFR*, for instance, *EGFR* mutations alone cannot account for all of the observed primary drug resistance in NSCLC patient groups. Therefore, we need to gain a better understanding of why these patients with hyperactive EGFR signaling have such variable responses to EGFR-targeted therapies by designing studies to explore variation in other non-*EGFR* genes. While we have primarily focused on somatic mutations and the differences in protein expression beyond *EGFR* that cause primary drug resistance to EGFR-targeted therapies, our findings extend beyond primary drug resistance; namely, the same genetic variation that is detailed in Table 1 might help to explain the rampant acquired resistance to EGFR-targeted agents that quickly follows an initial therapeutic response in a majority of patients with NSCLC who are treated with either erlotinib or cetuximab.

Deciphering the impact of mutations in, and variable expression of, non-*EGFR* genes on EGFR-targeted NSCLC treatment has significant and immediate clinical applications. At the prescriber level, one potential way to address the variations in other RTKs or in downstream pathway members of EGFR is to implement combination therapy. Given the relatively small number of RTKs in the human genome, more extensive genotyping can be performed to screen for the common activating mutations in non-EGFR RTKs. This would guide EGFR-targeted therapy choices and options for combination therapy. Thorough RTK screening could reveal mutant *RTKs* with oncogenic phenotypes that are silent or masked by the presence of the more aggressive mutant *EGFR*. If mutations are found in multiple RTKs, combination therapy approaches that target all identified RTKs could be a powerful way to minimize the risk of acquired resistance to EGFR-targeted therapy.

For instance, EGFR-targeted inhibitors could be coupled with inhibitors of KRAS, MET, PI3K, IKK, HER2, HER3, or even with inhibitors of HER2/HER3 dimerization, to anticipate resistance mediated by genetic abnormalities in members of these downstream or parallel pathways. In fact, several MET inhibitors are in phases II and III clinical trials in the United States, and some are even being examined in combination therapy regimens along with EGFR TKIs such as cetuximab [1,110,111]. The development of additional dual-therapy approaches that investigate the benefits of EGFR-targeted therapy in combination with other RTK inhibitors and downstream pathway inhibitors is in progress, and some combinatorial regimes show clinical promise over EGFR inhibitors alone [112,113]. It is important to note, however, that many of these new drugs are recent discoveries and are still in clinical trials. If and when these drugs are released to the market, the cost, adverse effects, and increased risk of drug-drug interactions versus potential clinical benefit should be taken into account during the process of deciding whether to administer combination therapy in the setting of NSCLC.

Even though the targeting of NSCLC with combination therapy is proving to be a powerful approach in patients whose tumors are resistant to EGFR inhibition, the mixed clinical results indicate that patient responses to combination therapies can be just as varied as the responses to EGFR inhibition. A more comprehensive understanding of the genetic landscape of NSCLC subtypes is thus needed to elucidate modulators of both drug resistance and drug sensitivity to the various combinations of targeted therapies. Therefore, while the pharmacogenomic associations listed in Table 1 provide valuable insight into the treatment of NSCLC, additional genetic factors will inevitably become important in the future as specific, multifaceted therapies are tailored to individual patients.
